# Leptospirosis in Squirrels Imported from United States to Japan

**DOI:** 10.3201/eid1207.060370

**Published:** 2006-07

**Authors:** Toshiyuki Masuzawa, Yoshihiro Okamoto, Yumi Une, Takahiro Takeuchi, Keiko Tsukagoshi, Nobuo Koizumi, Hiroki Kawabata, Shuji Ohta, Yasuhiro Yoshikawa

**Affiliations:** * Chiba Institute of Science, Choshi, Japan;; † Azabu University, Sagamihara, Japan;; ‡ Shizuoka Saisei-kai General Hospital, Shizuoka, Japan;; § National Institute of Infectious Diseases, Tokyo, Japan;; ¶ Tokyo Quarantine Station, Kawasaki, Japan;; # The University of Tokyo, Tokyo, Japan

**Keywords:** Exotic animals, *Leptospira*, Leptospirosis, Southern flying squirrel

## Abstract

We diagnosed leptospirosis in 2 patients exposed to southern flying squirrels imported from the United States to Japan. Patients worked with exotic animals in their company. *Leptospira* isolates from 1 patient and 5 of 10 squirrels at the company were genetically and serologically identical and were identified as *Leptospira kirschneri*.

Leptospirosis is a worldwide zoonosis caused by infection with *Leptospira interrogans* sensu lato species. *Leptospira* is mostly transmitted to humans through contaminated water or soil and by direct contact with a variety of infected animals (*1**–**3*). To date, a variety of wild animals have been imported from foreign countries to Japan. In this study, 2 men working at an animal trading company were infected with *Leptospira* spp. To determine the source of infection, *Leptospira* spp. were isolated from animals in their company and sequenced.

## The Cases

An animal trading company in Shizuoka, Japan, imported 106 southern flying squirrels from Miami, Florida, on March 27, 2005. Three workers handled these animals, which were housed 10 animals to a cage. Before patient 1 became ill, the workers dressed casually and touched the animals with bare hands in their routine work. Wild rats (such as *Rattus norvegicus* or *R. rattus*) had not invaded the animal house.

On April 22, 2005, patient 1, a 29-year-old man who handled a variety of exotic animals at the company, was hospitalized in Shizuoka Saisei-kai General Hospital with fever (temperature 40°C), headache, chills, nausea, vomiting, jaundice, and uremia, symptoms similar to those of locally acquired leptospirosis. Leptospirosis was diagnosed by polymerase chain reaction (PCR) targeted to the flagellin gene (*flaB*) and confirmed serologically with convalescent-phase serum by microscopic agglutination test. The patient was seronegative and PCR-negative for hantavirus, which causes symptoms similar to those observed in the patient. He was treated with an intramuscular injection of streptomycin (2 mg/day) for 7 days, which is the recommended treatment for leptospirosis in Japan (*4*); he consequently recovered.

On June 1, 2005, patient 2, a 28-year-old man who worked at the same company, was hospitalized in Shizuoka Saisei-kai General Hospital with fever (temperature 39°C), headache, chills, nausea, vomiting, jaundice, and uremia. The patient had been in contact with imported animals. He recovered with intramuscular injections of streptomycin (2 mg/day) for 3 days, followed by treatment with oral amoxicillin for 3 days.

*Leptospira* DNA was detected in serum samples from patient 1 and whole blood from patient 2 by *flaB* PCR (*5*). Sequences were determined by Prism 3130-avant DNA Genetic Analyzer (Applied Biosystems, Foster City, CA, USA). Sequences of *flaB* detected from both patients were identical and showed a high degree of similarity to *L. kirschneri*.

Diagnosis was performed serologically by microscopic agglutination test with a panel of *Leptospira* reference strains (*3*). Convalescent-phase serum samples from both patients reacted to *L. kirschneri* strain Moskva V and strains isolated from southern flying squirrels, although serum collected on the day of hospitalization was negative in both patients (Table 1). To cultivate *Leptospira*, a few drops of blood from patient 2 were placed in several tubes of Ellinghausen-McCullough-Johnson-Harris medium supplemented with 2.5% rabbit serum. After 7 days of incubation at 30°C, *Leptospira* was detected from the culture (isolates P5.4, P10.1, P10.2).

**Table 1:** Microscopic agglutination titer of patients' sera collected while hospitalized and during the convalescent phase

Patient	*Leptospira* strain used as antigen*	Patient serum	
		Hospitalized	Convalescent-phase
1	Serovar Grippotyphosa Moskva V	<50	100
	Animal isolate AM3	<50	800
	Animal isolate AM1	<50	800
2	Serovar Grippotyphosa Moskva V	<50	200
	Animal isolate AM3	<50	200

*Samples were not reactive to a panel of representative serovars, Australis, Autumnalis, Carlos, Bataviae, Cynopteri, Hebdomadis, Copenhageni, Icterohemorrhagiae, Javanica, Pomona, Pyrogenes, Hardjo, Sejroe, Wolffi, and Tarassovi; serovars Canalzonae, Huanuco, Muelleri, and Valbuzzi belong to serogroup Grippotyphosa.

**Figure 1:**
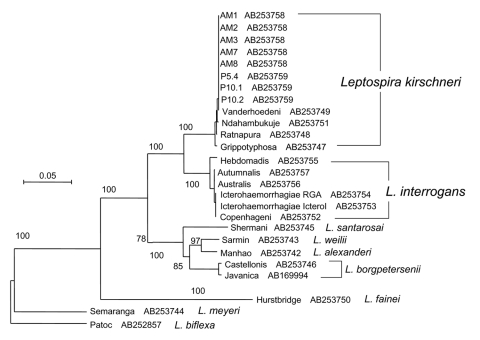
Phylogenetic tree based on the *Leptospira* DNA gyrase B subunit gene (*gyrB*) sequence. The sequences obtained have been deposited in DDBJ/GenBank/EMBL with accession numbers indicated.

**Figure 2:**
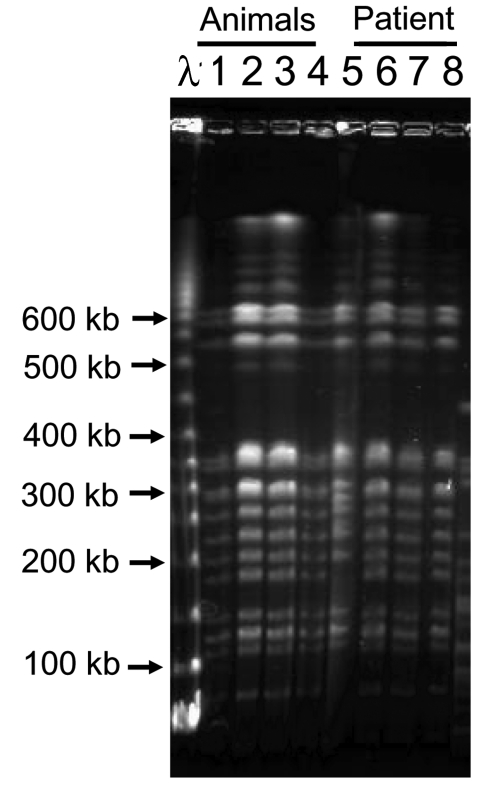
Pulsed-field gel electrophoresis analysis of *Not*I restriction fragment of *Leptospira* isolates from patient 2 and southern flying squirrels. *Leptospira* cells were lysed, and DNA was digested with restriction enzyme *Not*I in agarose gels. DNA in the gel was electrophoresed with 1% pulsed-field certified agarose in 0.5× Tris-borate-EDTA buffer under a pulse time of 10 s for 5 h and 30 s for 12 h, followed by 60 s for 7 h at 200 V. Lane 1, AM1; lane 2, AM2; lane 3, AM3; lane 4, AM7; lane 5, serovar Grippotyphosa strain Moskva V; lane 6, P10.2; lane 7, P10.1; lane 8, P5.4; λ phage DNA concatemer is used as a DNA size marker. Isolate AM8 showed an identical restriction fragment length polymorphism pattern to that of others.

To determine the validity of the association between animals held by the company and the illness, exotic animals (75 animals, 7 species) housed in the company were tested. *Leptospira* was isolated from 5 of 10 kidney cultures (isolates AM1, AM2, AM3, AM7, AM8) from southern flying squirrels. DNA from the urinary bladders, including the animals' urine, was extracted by using proprietary DNA extraction kits (Quick gene, Fuji Film Co., Tokyo, Japan). Five of 10 southern flying squirrels were *flaB* PCR-positive (Table 2). Species of the isolates were identified by using *flaB* and DNA gyrase B subunit gene (*gyrB*) sequencing analysis. We amplified 1.2-kb partial sequences of *gyrB* by using primers UP1TL (5´-CAyGCnGGnGGnAArTTyGA-3´; n: A, G, T, or C; r: A or G; y: C or T) and UP2rTL (5´-TCnACrTCnGCrTCnGTCAT-3´; n: A, G, T, or C; r: A or G) (*6*). The isolates obtained from patient 2 and southern flying squirrels had identical *flaB* (data not shown) and *gyrB* (Figure 1) DNA sequences and were identified as *L. kirschneri.* The *flaB* sequences from the serum of patient 1 and whole blood of patient 2 were identical to those of isolates from patient 2 and animals. Additionally, restriction fragment length polymorphism (RFLP) analysis based on pulse-field gel electrophoresis was conducted (*7*). These isolates showed identical RFLP patterns (Figure 2), which suggests that patients were infected with *L. kirschneri* from southern flying squirrels.

**Table 2:** Detection and isolation of *Leptospira* from imported animals in the company

Animal	No. samples positive/no. samples tested	
	Kidney culture	*flaB* PCR
Spiny mouse (*Acomys cahirinus*)	0/9	0/9
House mouse (species unknown)	0/4	0/4
Golden spiny mouse (*Acomys russatus*)	0/13	0/13
Mongolian gerbil (*Meriones unguiculatus*)	0/9	0/9
Southern flying squirrel (*Graucomys volans*)	5/10*	5/10*
Baluchistan pygmy jerboa (*Salpingotulus michaelis*)	0/20	0/20
Siberian chipmunk (*Tamias sibiricus*)	0/10	0/10

*Four of 5 culture-positive animals were positive by polymerase chain reaction (PCR). Remaining culture-positive animal was PCR negative, whereas 1 culture-negative animal was PCR positive.

To determine serovar of the isolates, a cross-agglutination test was performed with a panel of hyperimmune rabbit serum raised to representative serovars Icterohemorrhagiae, Copenhageni, Autumnalis, Hebdomadis, Australia, Grippotyphosa, Javanica, and Castellonis, which are present in Japan. These isolates reacted with anti-Grippotyphosa serum but not with the others (data not shown). Convalescent-phase sera from patients reacted with *Leptospira* isolates from the squirrels and also with serovar Grippotyphosa strain Moskva V (Table 1).

On April 24, the local health government prohibited the company from trading animals and directed them to use protection, such as latex gloves and disinfection of the floor with sodium hypochlorite, against infection. On June 2, all southern flying squirrels were euthanized by carbon dioxide, and the animal house was disinfected by the local health government. PCR detected *flaB* DNA on the surface of the squirrels' bodies and in urine on the soaked paper in the cages; the sequences were identical to those of the isolates. Before the first case was detected, 27 southern flying squirrels had been distributed to retail pet shops. Sixteen were returned, 2 died, 7 remained at pet shops, and 2 had been sold. The 2 sold animals and 7 remaining at the pet shops were recovered and euthanized. No illness was reported among persons in contact with these animals.

## Conclusions

Serovar Grippotyphosa commonly causes canine leptospirosis (*8**,**9*) and infects a variety of domestic and wild animals in the United States (*10**–**13*). In Japan, serovar Grippotyphosa is distributed in the southernmost islands, the Okinawa archipelago (*14*), but not on Honshu Island, the main island. Patients did not travel to Okinawa or foreign countries before disease onset. Our findings support the conclusion that the patients were infected with *L. kirschneri* serovar Grippotyphosa by contact with southern flying squirrels. Similarly, in the United States, humans have acquired monkeypox infection from pet prairie dogs, which had themselves been infected by exotic African rodents (*15*); these findings show that exotic pets represent a substantial hazard. The outbreak demonstrated how new infectious diseases could be emerging because of importation from overseas. If, during shipping and housing of the animals, the infection were to have expanded among southern flying squirrels, the infection rates and risk for humans would have increased. The leptospirosis cases reported here warn against importing exotic animals.
